# Extra Supplement—The Nursing Mirror

**Published:** 1890-10-04

**Authors:** 


Being the Extra Nursing Supplement of "The Hospital" Newspaper.

All Contributions for this Supplement should be addressed to the Editor, The Hospital, 140, Strand, London, W.C., and should have the word "Nursing" plainly written in left-hand top corner of the envelope.

### En Passant.

NURSING IN INDIA.—To be invalided in India will now be a much slighter hardship than it was formerly, thanks to the successful working of Lady Roberts' scheme for providing trained lady nurses in the military hospitals there. The Quetta Home (built by private subscription) is open, and three nurses are already there. The diminished death-rate in the military hospitals where trained nurses are employed, and the appreciation which the soldiers show for their extra comfort, must be very gratifying to Lady Roberts.

THE ADVANCE OF THE PRIVATE NURSE.—Miss Hooper has sent us her register of nurses, giving particulars of the training and qualification of those women whose names are on her books. This is a move in the right direction, but we should have preferred even fuller particulars. Miss Hooper charges a small registration fee, and a commission of two shillings in the pound on all cases introduced by her agency. We also hear that the Nurses' Co-operation is growing in strength and power, and will shortly come before the public with a carefully prepared scheme, which has been worked out by a committee during the last year. The Co-operation is founded on the lines of the American Directories for Nurses.

SOLDIERS' WIVES AS NURSES.—Arrangements have been made at Aldershot for soldiers' wives to undergo a course of training in midwifery and general nursing, in a class to be formed on the 15th proximo, under the direction of Surgeon-Major Finlay, of the Army Medical Department. Since soldiers' wives often accompany their husbands to out-of-the-way places where nurses are not, this move is certainly a good one. The only thing is, are the pupils to have clinical instruction? Otherwise mere lectures are comparatively useless.

SHORT ITEMS.—Six hundred midwives attended the congress at Berlin last week.—On September 18th, Dr. Francis Heatherley, of New Ferry, was married to Miss Grace E. Sackett, recently matron of the Stratford-on-Avon Hospital.—A correspondent writing of the Levick Institution claims for another Paris Home the title of Chief Institution under a trained hospital nurse; unfortunately, our correspondent forgets to give the name and address of this old institution.—Nurse Garside writes to say what a pleasant holiday home she found at Scarborough with Sister Dora.—Lady Roberts returns to India next spring, and will visit the new Quetta Nursing Home.—The nurses at Hanwell are to be provided with a piano and other means of amusement to relieve the dull monotony of their lives, and tempt them to remain longer at the schools.—Miss S. A. C. Reedham, certificated nurse, has had an action for libel brought against her at Rochdale; she had to apologise and pay the costs.—The matron of the London, in her opening lecture to the new probationers, strongly urged them, if not strong enough to sacrifice the comforts of home life, to return to their homes, and not stay and injure the hospital.—Miss Lückes writes an article in the *New Review* on "Trained Nurses at the London Hospital."

HOME OF REST AT BOGNOR.—Among the many Homes of Rest for ladies of small means there is one at Bognor which was started two years ago—in the summer of 1888—by a lady entirely on her own responsibility, and she devotes herself to its superintendence. It is not intended for invalids so much as for ladies who from delicate health or overwork require rest and sea air at a small cost. The two houses are connected on the ground floor by a passage. There is a bright, pleasant drawing-room, with a cheerful outlook, and it is only a short distance from the sea, while quite near there are pleasant country walks. It is pre-eminently a place of rest; but as a trained nurse resides in the home, there is always help at hand in case of illness, and the Lady Superintendent is experienced in nursing. There are beautiful drives in the neighbourhood, and among other places of interest Arundel, with its grand old castle and its park.

IN CALIFORNIA.—Those who have been travelling during their autumn holidays must have been struck by the ubiquitousness of the trained nurse: she is everywhere. In Jerusalem or Denver, in Canary Isles or Central Russia, you find the trained nurse is now to the fore. When the North Pole is reached we are confident a trained nurse will be found there busily engaged in attending the sick polar bears. Our latest foreign correspondent writes from San Diego, the town with a bay and a climate, as she calls it. The bay is very fine, the climate delightful, and the city is thriving, having well-paved streets and splendid free schools. There is a Cottage Hospital belonging to the Episcopal Church, only eighteen months old; the matron trained at Albany. The "Sisters" have just started a hospital on the first and second floor of an hotel; no doubt they will do well, as they are much liked and respected. The very poor sick are taken to the "Poor Farm or Country Hospital," some few miles from the town, which consists of four or five long low buildings; there are no nurses, though there are often 90 patients. The private nurses work on their own account, and are not trained—only experienced. We hope to hear more of life in California from this correspondent later on.

MIDWIFE OR CHARWOMAN?—The supporters of the Midwives Registration Bill may find food for thought in the following case: "Bowerman v. Reynolds." The plaintiff, a nurse, of Twickenham, summoned the defendant, a gentleman, of 14, Amyand Park-road, Twickenham, for £3 3s. on account of an alleged engagement as monthly nurse. Mr. Perry, solicitor, represented the plaintiff, and Mr. Lay, solicitor, the defendant. The plaintiff's case was that she was suddenly summoned to the defendant's house for the purpose of attending his wife in an expected confinement. The summons was premature, but she continued in the house for some days, and subsequently left some of the things at the house and held herself in readiness to go there again when required at Mrs. Reynolds' request.—The defendant stated that when she asked Mrs. Bowerman whether she could attend her, she said that she would do so at that particular time, but could not engage herself as monthly nurse, because she was liable to rheumatism in her hands. She was employed in the house for a few days, but only as a charwoman, and was paid as such. Some conflicting evidence having been given, his honour found for the defendant.

### The Lifting and Moving of Patients.

In these days of trained nursing, when the clearest brains and the deftest fingers are not always allied with the greatest muscular strength, it is well that nurses, more especially those engaged in private work, should remember that, as in most things, there is an easy and a difficult way of moving and lifting patients. Most of us have noticed with what ease and comfort some fragile-looking woman will manage, while another of far greater bulk and strength will tug and strain till both herself and the invalid are in a state of heat and exhaustion. Nothing but experience and practice will give the needed dexterity, but perhaps a few practical hints on this and kindred subjects may be useful.

In the first place the bedstead should be narrow, that the patient may be easily reached from either side; the mover loses half her power in leaning over to move an invalid who is sunk in the middle of a large bed. Sometimes in private nursing this inconvenience has to be put up with, and when this happens one side should be used for day and the other for night; the unoccupied half being thoroughly aired by folding back the bed clothes into the middle of the bed and allowing them to remain for a time. The intelligent nurse before lifting her patient will see that the way is clear, that there are no lumps or ridges in the bed to impede her movements, that the bed-clothes are folded back so that they may not cling to the patient and increase the weight. She will have the fresh side of the bed or the couch prepared beforehand, the mattress and the pillows so arranged that the patient may be deposited comfortably and need little disturbance after. Nurses need scarcely be told that neither crumbs nor wrinkles should be allowed in the sheets; if they are narrow or short keep them taut by fastening them to the mattress with large safety pins or needle and thread. A feather bed adds greatly to the difficulty of moving a patient and for this and other reasons should never be used. In moving an invalid from one side of a double bed to the other, the nurse should stand on the side nearest to him and placing her hands beneath the shoulders and thighs, lift him from her to the middle of the bed, then moving to the opposite side she should lift him towards her in the same way. If the patient is heavy, and help cannot be obtained, it is better to pass a draw-sheet beneath him, and to move him from one half of the bed to the other by means of firm, slow traction on the pillow and draw-sheet, this is much less fatiguing to the patient than inefficient lifting. Two persons should lift by joining hands beneath the shoulders and thighs of the invalid, and in cases of fracture a third person is required to steady the injured limb. In carrying a patient who is allowed to sit upright, two nurses should make a sedan-chair by each clasping her right wrist with her left hand, and her companion's left wrist with her right hand; the patient being seated on the side of the bed or couch, the hands and wrists join beneath his thighs; he then places his arms around the nurses' necks to keep in position, and can be easily carried. In moving an invalid from the bed to the couch the latter should be placed side by side with the bed and made level with it by means of mattresses and pillows. In placing a bed-pan beneath the patient the nurse having first seen that the legs are drawn up, should lift, if possible, with her right hand under the lower part of the patients back, putting the pan under with the left—the right hand and arm being usually the stronger. With heavy female patients, where help and conveniences were hard to obtain the writer found a flat-edged pie-dish much easier to use than the ordinary bed-pan or slipper, it does not require to be placed so far under; of course the nurse will destroy the utensil when no longer required. In spinal and some other cases where the invalid is allowed to be carried into the garden or on to the shore, a stretcher should be provided. If a pair of light poles, similar to broom-handles but longer, can be obtained, it can be easily made under the nurse's supervision. The body is made of bed-ticking or any strong material wide enough to allow a broad hem on each side; this is placed under the patient, and the poles then slipped through the hems, a piece of each pole projecting beyond the material to form the handles. A carpenter or handy-man will make the holes and the spars at top and bottom. The writer once made an impromptu stretcher out of a pair of prong handles and a piece of old quilt; strips of thin board lodged across the top and bottom prevented the patient from getting pressed. In spinal cases it should be remembered that the least jar may cause agony; all movements should be gentle and gradual. In cases of typhoid, hæmorrhage, and heart disease a sudden movement may have dangerous consequences. When advisable to keep the patient as still as possible, the night-gown should be slit up the back and thus easily changed. When there is abdominal pain or tenderness prop up the knees with a pillow, that the tension and consequent pressure may be lessened. A change of posture will often relieve pain; always move a patient if he complains of numbness or tingling in a limb, it is generally caused by pressure on a nerve or blood vessel. While attending to bed sores or washing the back, the nurse should see that the invalid is comfortably supported by one or more pillows. When the patient is propped up by bed rests or bolsters take care that he is not continually slipping down; this may be prevented by placing small blocks of wood or old books under the bottom feet of the bedstead; in some cases a spare bolster placed under the mattress and pushed up close to the buttocks is the greatest possible comfort. A chair turned bottom upwards and arranged with pillows is a good substitute for a bed rest. A fractured limb should be lifted by placing the hands under the limb above and below the fracture. A patient with a broken limb should not be turned on his side; his sheets should be changed from the head downwards, the shoulders being first raised while the sheet is drawn under down to the small of the back, then raise the lower part of the body, and lastly the feet. In moving those patients who wear instruments, care should be taken that small pieces of skin are not nipped between the instrument and the nurse's hand. In many cases nervous patients should be encouraged to help themseles as much as possible, but great judgment is required, as some of them will make efforts beyond their strength and then collapse for days. After a long illness, it will often be found that joints are stiff and muscles contracted through want of use and lying in one position. Usually the services of a skilled masseuse are called in, but the nurse may do much, with the doctor's sanction, to prevent or remedy the contractions. A few simple and easy joint exercises should be gone through daily as soon as the invalid's strength permit. To exercise the legs place one hand beneath the patient's knee and let his heel rest in the hollowed palm of the other, bring the heel gently towards the thigh, at the same time raising the knee; repeat this several times and finish by slowly rotating the limb. For the arms, take the elbow in the hollow of one hand and grasp the wrist with the other, bring the patient's hand to his shoulder and the elbow to his side; straighten the arm outwards and upwards; repeat and rotate. In moving the finger-joints in rheumatic subjects, the patient's hand should rest on a firm pillow; each phalange must be exercised separately. In practising the exercises the grasp of the nurse should be firm and decided, and the movements slow and even. Jerks and half-hesitating touches will irritate the most amiable of patients. It is well to remember that the knee, elbow, wrist, ankle, fingers and toes, have hinge-joints, and can only move backwards and forwards; while the hips and shoulders have ball and socket joints, which allow of movement in all directions. The thumb has a double joint, and moves from side to side as well as backwards and forwards; while the radius or small bone of the arm moves round on the larger by means of a pivot joint, and this allows the upwards and downwards movements of the palms of the hand.

### Six Months in a Hospital.

(By a "Special Pro.")

*(Continued from page cx, Vol. VIII.)*

II.

My Dearest Cassy,—Thank you very much for your kind little note; it got here before I did, and when the maid opened the door to me she gave it me. It felt like being at home! This is a funny old Hospital; very much on the "rough and ready" system—meal times in particular, but the nurses are all exceedingly kind. *Nurse Stewart* (!) and Nurse White, are the only "Specials" here. We have a room together at the top of the house, away from the dormitories, and a fire every night to go to bed by. We have two beds, two looking glasses, two recesses for our clothes, indeed, two of everything, so we can't quarrel! There are two tiny sitting rooms for the nurses' day rooms, one for the staff and "specials" the other for the regular "pros." There is certainly nothing very aristocratic here, and I don't know yet whether I should like it enough to fix down for three years. We have 16 beds in my ward; a staff nurse, an assistant nurse and a "pro."—myself! I have had scarcely anything to do yet, except carry a few lunches, etc.; and a little light work. I suppose I shall have more later on. I am in a male medical ward, but we have surgical cases too. Our last arrival was a pleurisy patient, who is very bad, poor fellow! I gave him some medicine, and, of course, spilt it down his shirt! Like a probationer wasn't it? Poor thing! I shouldn't like to be practised upon by ignorant beginners, would you? We get up at seven (I and my fellow "special"), breakfast at a-quarter to eight; go on duty at half-past eight; come off from ten to twelve each day. On Sundays we have from ten to one, and from two to four, so it is a light day for us; then we have a whole day once a month as well. My staff nurse, C. D., is very kind. She is a funny-looking, little woman, dark, with bright eyes. I am very glad I have come, although I have not been here long enough to get into the spirit of the work yet.

I feel exceedingly awkward and uncomfortable at intervals, but I have had some good laughs since I came! All the staff got into a row with the matron last night for warming themselves at the dormitory fire ten minutes after half-past ten. She pounced down upon them unexpectedly, and they were having immense fun over it to-day. They say now that they won't be able to go to heaven soon unless their passports are signed by Miss J——.

I am beginning to feel more at home here, and much more comfortable, although the grammar of some of my fellow nurses is continually giving shocks to my nervous system! I have been trotting all over the hospital lately doing relieving duty, and have been in nearly every ward.

My last two days were spent in the "isolated" ward with one of our nurses, who had knocked herself up altogether, and they didn't know what it was. She is now, much to her disgust, transferred to a general ward, and I am back in my own. I am sorry to tell you that in the course of my recent relieving duty the last three babies I have assisted to nurse have all died. One was a most interesting case—brain disease, fits, &c. Such a dear little pretty baby! Its mother used to sit up with it all night at the last.

I saw a woman "tapped" the other day; never saw it before, and was much interested.

One of my few "friends" here, H. W., a doctor's daughter, a very High Church girl, is taking her training here in order to give herself up afterwards entirely to nursing the sick poor in connection with a church, or to join some "order."

At least so she purposes at present; but what are our purposes compared with our circumstances, and the dreadful doctrine of philosophical necessity? I am going to read a a novel! I know it is a new feature in my case; nevertheless, it is true. What would you advise? I give you until June 2nd to decide, as my "physiology" examination is on that day, and after that I mean to begin. I am perfectly sick of cramming up bones and nerves, and must have different diet for a time.

I have been to several operations, one a tumour, back of neck; varicose vein in leg, hæmorrhoids, etc., and haven't felt the least bit upset, although I admit they are rather "creepy" performances! The only thing that nearly did upset me (although not outwardly) was a burn case, in a ward in which I was assisting for the day. It was really too horrible to describe; the woman shrieked at the top of her voice the whole day except when under morphia. I had to help hold her up during the dressing (it took three people to do it), and I could not get her shrieks out of my head for days. It is dreadful when people have no control over their feelings at all, but give way entirely; some are so different. In our ward there is nothing of that sort fortunately. We have rheumatics, paralysis, consumption, pleurisy, etc., but nothing very dreadful.

### Everybody's Opinion.

*[Correspondence on all subjects is invited, but we cannot in any way be responsible for the opinions expressed by our correspondents. No communications can be entertained if the name and address of the correspondent is not given, or unless one side of the paper only be written on.]*

### MALE NURSES.

"A Male Nurse" writes: It is decidedly amusing (except to those in question) to study the treatment of nurses of the Hamilton Association. A would-be nurse on joining has to answer the following questions in writing, and sign them:—

Question 11. Are you willing to undergo such a course of training as the committee may deem necessary in order that you may become a qualified nurse? (12) while undergoing such training are you prepared and willing to pay for your own board and lodging in the neighbourhood of the place where you will be trained? (13) what amount are you prepared to contribute towards the expenses of training?

He will procure at his own expense, and maintain in a respectable condition, a suitable outfit during training. When (not in a year, as recently stated) his progress and acquirements are proved satisfactory, a certificate of fitness as a trained male nurse may be granted him.

Every man on making application to be registered as a candidate for employment as male nurse, or to be trained as such under the Hamilton Association, will pay 20s. towards its funds and expenses.

Previous to being given employment, he must sign the rules and regulations in token that he understands them, and that he agrees to abide by them. He must at the same time deposit 40s. as caution money.

Another reads: Minor offences will entail a fine from 1s. to 40s. (according to the circumstances of the case), to be deducted from their wages.

Another: If any employer fails to pay the amount due for the service of any nurse, and in the opinion of the committee it shall not be thought desirable to take legal proceedings against such employer to recover the amount due, the association shall not be bound to take any such proceedings or make good any such loss to the nurse.

Nurses will not be allowed to take cases on their own account while on the roll of the H.A.

It will be seen by this selection that it is taking up the nurses' side of the association.

He must wait at home day and night until wired to, or sent for any length of time, often varying from a week to two and three months—that is a nurse's statement. He may not take employment if offered to him by other than his association. Why not make an affair like the Hamilton Association a limited company, shares being given to nurses only, and make them share profit and loss alike, and get cases for the company when not employed?

It is not a personal matter, but one can read "self" on the part of the association, and the nurse must take his chance. Fancy a married man employed three nights a week at an hospital at the rate of £1 a-week, and should his case die, or be well enough to dispense with a special nurse, he is thrown out at once.

On the other hand, who would wish for an annual pittance? The placing of the forms for application for enrolment into the hands of employers of nurses has not the tendency to raise the opinion of them with regard to nurses. Why not pay him interest on his deposit of caution money? Why report that fines were enforced in sixteen cases last year? They never ought to have been enforced. If a nurse breaks a rule, or misconducts himself, he ought to be dismissed or asked to resign, and not treated as if he were before the magistrates, where he certainly would get the benefit of an open court. Vale!

### Lectures.

THE lecture season is again commencing, and we call the attention of our readers to the following courses, for we strongly deprecate the habit of most nurses of letting the world and its work go by unheeded, save in the small sphere in which they minister. No greater rest is possible under mental strain than change of thought and scene and society.

TOYNBEE HALL.—On October 6th, at eight p.m., a course of lectures on "Electricity and Magnetism," by A. H. Fixon, D.Sc., will be commenced; fee, 1s. Other courses will be on "Dante," "The French Revolution," and "Wind and Water." A class on "Physiology" commences on October 6th. Every Saturday evening is a free popular lecture, and amongst this session's lecturers are Mrs. Fawcett and Professor Creighton. A stamped envelope should be sent to the secretary if further particulars or tickets are required.

GRESHAM HALL.—On October 7th, 8th, 9th, and 10th Dr. Symes Thompson will deliver, at six p.m., the Gresham lectures on "Physic," free to the public.

WORKING MEN'S COLLEGE.—Free Saturday evening lectures for men and women are delivered at half-past eight p.m., on October 11th by Churton Collins, October 18th by T. W. Wheeler, Q.C., on October 25th by Professor Bonney.

UNIVERSITY HALL, Gordon Square.—Lectures on "The First Three Gospels and the Early Church" will commence on October 8th; on "English Poetry of the Nineteenth Century" on November 4th.

Dr. Catherine Urquhart will commence a course of lectures on "Health and Sick Nursing" at Edinburgh in November.

Lecture programmes for the winter have been arranged by the Birkbeck and Westbourne Park Institutes.

### Appointments.

[It is requested that successful candidates will send a copy of their applications and testimonials, with date of election, to THE EDITOR, The Lodge, Porchester Square, W.]

LAMBERT MEMORIAL HOSPITAL, THIRSK.—Miss Bradbury, who trained at Leeds Infirmary, and has lately been working at the York Home, has been appointed matron of this new cottage hospital.

SEAMEN'S HOSPITAL, CONSTANTINOPLE.—Miss Emily F. Campbell sailed last week to undertake the post of chief nurse to this hospital. Miss Campbell trained at Edinburgh Royal Infirmary, Edinburgh Fever Hospital, and York Road Lying-in Hospital. For the last three years Miss Campbell has been nurse-matron of the Chesham Cottage Hospital. She holds excellent testimonials both as a nurse and as a woman, and we cannot but wish her a pleasant and a profitable time at her new post.

### Notes and Queries.

**Answers.**

(34) *Vanessa.*—Children's clothes would be most acceptable at Children's Home, Seal, Sevenoaks.

(38) If *Rix* will communicate or call on Thompson and Co., Pharmaceutical Chemists, Lodge Lane, Liverpool, S., they will be pleased to furnish full particulars where the course required may be obtained.

(39) *Nurse* had better not go to Bombay unless she knows doctors there. The climate is unbearable for part of the year, and all the residents go to the hills. There are several hospitals, notably the European General Hospital at Fort St. George, and the Jamsetjee Jejeebhoy Hospital at Byculla.—*Indiana.*

(40) *H. C. H.* had better apply to Messrs. Atkinson and Co., 198 to 212, Westminster Bridge Road, regarding patients' linen sheeting with two narrow stripes (red or blue).

*A stigmatism.*—We never give medical advice, or recommend particular practitioners. An oculist should be consulted, and your own medical man will give you the name of one.

*Sister Agnes.*—You can get the nurse's case you require from Down Bros., 3 and 4, St. Thomas's Street, Borough. Pardon delay in sending this answer.

### Vacancies.

THE following vacancies are announced:

**Nurses.**

Ashburton and Buckfastleigh Cottage Hospital; £25.

Bolton Fever Hospital; £25.

Borough Fever Hospital, Sheffield; £25.

Bournemouth Institute; £25.

Borough Hospital, Birkenhead; £25.

Blackheath and Richmond Institutions.

Brixton Institute.

Buckinghamshire Infirmary; £25.

Bedfordshire Hospital Institute.

Cheltenham Institute.

Cheltenham Institution.

Cambridge Home.

City and County Institution, Worcester.

Chest Hospital, Victoria Park, E.; £20.

Croydon Institute.

Devon and Exeter Hospital (Assist); £18.

Easter Ross Combination Poor House; £30.

Guest Hospital, Dudley; £25.

Also Night; £22.

Hampstead Home Hospital; £20.

Holborn Union Infirmary (Assist.); £17.

Hanover Institute, Hanover Square, W.; £25.

Isle of Wight Infirmary (Assist.); £18.

Liverpool Institution (male and female); £25.

Leeds Hospital for Women (night); £20.

Leeds General Infirmary (Supt.); £50.

Liverpool Stanley Hospital; £25.

Nurses' Home, Hull.

Nurses' Home, Sheffield.

Nurses' Home, Norwich.

Nursing Institute, Henrietta Street, W.

Oswestry and Ellesmere Cottage Hosptial; £25.

Poplar Hospital, E.

Royal Infirmary, Newcastle-on-Tyne; £20.

Royal Hospital, Belfast (night); £25.

Southport Infirmary; £25.

St. Helena Home, N.W.

St. Saviour's Infirmary, S.E.; £20.

Surrey County Asylum, Brookwood; £16.

St. Cuthbert's Hospital, Edinburgh; £25.

The Sanatorium, Hull; £30.

The Sanatorium, Margate; £24.

Victoria Home, Bournemouth; £25.

Wilson Institution, Wimpole Street, W.

Workhouse Infirmary Nursing Association.

Worthing Infirmary; £24.

Wirral Children's Hospital, Birkenhead.

**Probationers.**

Boston (Lincs.) Hospital.

Hospital for Consumption, Ventnor, I.W.; £10.

Leeds Institution; £14.

N.C.C. Hospital, Wisbech.

Poplar Hospital, E.

Princess Alice Memorial Hospital, Eastbourne.

St. Helena Home, N.W.

Woolwich and Plumstead Cottage Hospital.

**District Nurses.**

Bristol Society; £42.

Leeds Home; £25.

South London Association.

**Matron.**

Engedi Convalescent Home, Brighton.

### Amusements and Relaxation.

**N.B.—THIRD QUARTERLY WORD COMPETITION**

**Commences Oct. 4, 1890; ends, Dec. 27, 1890.**

Three prizes of 15s., 10s., 5s., will be given for the largest number of words derived from the words set for dissection.

N.B.—Word dissections must be sent in WEEKLY not later than the first post on Thursday to the *Prize Editor*, 140, Strand, W.C., arranged alphabetically, with correct total affixed.

The word for dissection for this, the FIRST week of the quarter, being

"SURGEON."

| Names. | Sept. 25th. | Totals. | Names. | Sept. 25th. | Totals. |
|---|---|---|---|---|---|
| Dulcamara ......... | — | ... 503 | Jenny Wren ...... | 184 | ... 766 |
| Tinie .................. | 208 | ... 830 | Rhoda ................ | — | ... 242 |
| Henri.................. | — | ... 395 | Annabel ........... | — | ... 17 |
| Agamemnon ...... | 217 | ... 850 | Spare Moments ... | — | ... 19 |
| Patience ............ | 209 | ... 832 | Qu'appelle ......... | 191 | ... 801 |
| Brisbane ........... | — | ... 332 | Nurse.................. | — | ... 29 |
| Nurse Hilda ...... | 172 | ... 772 | A. B. C................ | — | ... 251 |
| Lightowlers ...... | 201 | ... 818 | Brickbat ........... | — | ... 77 |

**Notice to Correspondents.**

Qu'appelle.—Sept. 18th, 23.

